# Complementary Feeding and Infant Gut Microbiota: A Narrative Review

**DOI:** 10.3390/nu17050743

**Published:** 2025-02-20

**Authors:** Danielle L. Noles, Kinzie L. Matzeller, Daniel N. Frank, Nancy F. Krebs, Minghua Tang

**Affiliations:** 1 Department of Pediatrics, Sections of Gastroenterology, Hepatology, and Nutrition, University of Colorado Anschutz Medical Campus, Aurora, CO 80045, USA; danielle.noles@childrenscolorado.org; 2 Department of Pediatrics, Section of Nutrition, University of Colorado Anschutz Medical Campus, Aurora, CO 80045, USA; kinzie.matzeller@colostate.edu (K.L.M.);; 3 Department of Food Science and Human Nutrition, Colorado State University, Fort Collins, CO 80526, USA; 4 Department of Medicine, Division of Infectious Diseases, University of Colorado Anschutz Medical Campus, Aurora, CO 80045, USA

**Keywords:** complementary feeding, gut microbiota, infant feeding, nutrition

## Abstract

**Background**: The complementary feeding period, spanning from 6 to 24 months of age, marks the transition from an exclusive liquid diet in infants to a dietary pattern requiring the introduction of solid foods to meet nutritional demands. Complementary feeding coincides with other critical development windows, including the maturation of the gut microbiome. However, the effects of specific solid foods on gut microbiota and the subsequent influence on health outcomes require further investigation. **Methods:** This narrative review analyzes published research from January 2004 to October 2024 and aims to summarize the current evidence of the effects of complementary feeding on the infant gut microbiota. **Results:** A total of 43 studies were included in this review. Overall, multiple studies reported an increase in alpha-diversity after solid food introduction. *Bifidobacteriaceae* is the predominant bacterial family during the first 6 months of life, shifting to *Lachnospiraceae*, *Ruminococcaceae*, and *Clostridium* spp. after the introduction of solid foods. The timing of solid food introduction may also influence gut microbiota, though results were inconclusive. The effect of individual dietary components on the gut microbiota was conflicting, with limited evidence to make inferences. **Conclusions:** Because of variations in study design, dietary intake quantification, and minimal follow-up, a lack of conclusive evidence exists describing the relationship between complementary feeding and gut microbiota outcomes in infants. Future research to describe these relationships should focus on the impact of individual foods on microbial diversity and maturation, as well as the relationship between microbiota and infant health outcomes.

## 1. Introduction

The complementary feeding period (~6 to 24 months), when infants start to consume foods beyond breastmilk or formula, represents significant changes in infants’ diets from the liquid diet phase (breastmilk and/or infant formula) to the introduction of solid foods and changes in nutrient and calorie intakes. Undesired growth patterns during infancy, namely rapid or excessive weight gain and/or fat gain relative to length, are strongly associated with childhood obesity [1,2,3]; additionally, growth restriction and growth faltering during this time have long-term consequences on health outcomes in infants [4]. Complementary feeding is emerging as a contributing cause of weight gain in infancy and is associated with rapid infant growth, a main driver of obesity and metabolic syndrome development in adolescence and adulthood [3,5,6]. The complementary feeding period also entails drastic changes in the gut microbiome, compromising both the host intestinal tract and its resident microorganisms (i.e., the gut microbiota). Research in low-resource settings has demonstrated that manipulating the gut microbiota during the complementary feeding phase could reduce stunting in infants and toddlers [7,8,9]. This has prompted the question of whether the gut microbiota could also be manipulated to reduce obesity and metabolic syndrome. However, before effective interventions can be implemented targeting the gut microbiome to prevent rapid weight gain and obesity development, it is critical to evaluate the current evidence on how complementary feeding affects infant gut microbiota.

Although the gut microbiota is greatly influenced by diet, we know very little about the effects of complementary feeding on its development. The consensus is that the introduction of complementary foods increases microbial diversity and reduces the abundance of certain taxa, such as *Bifidobacterium* [10]. More research, though minimal, has been published over recent years reporting the introduction of various complementary foods and the timing of their introduction on the gut microbiome. Thus, the objective of this narrative review is to summarize the current evidence supporting the notion that complementary feeding has demonstrable and biologically meaningful effects on infant gut microbiota and the impact that the timing of solid food introduction may have on observed effects.

## 2. Materials and Methods

### 2.1. Information Retrieval and Data Source

The online databases Web of Science and PubMed were searched to identify papers relevant to the research question. Search terms included “microbiome” OR “microbiota” “complementary feeding” OR “complementary foods” OR “complementary diet” OR “complementary feedings” OR “solid food” OR “solid foods” OR “infant diet”, AND infant or infants. With the added MESH term in PUBMED “microbiota”. Articles were reviewed from January 2004 to October 2024.

### 2.2. Inclusion and Exclusion Criteria

Prior to searching for articles, inclusion and exclusion criteria were established. Human qualitative and quantitative studies related to complementary feeding and the infant gut microbiome were included in the review. Complementary feeding was defined as the introduction of solid foods during the first year of life. To minimize selection bias, two reviewers, D.N. and M.T., independently reviewed the articles to evaluate their relevance to the review topic. Articles were excluded if they did not pertain to complementary feeding or solid food introduction and the infant gut microbiome. Study protocols, non-human studies, proof of concept studies, in vitro studies, and studies specifically evaluating non-food items (e.g., vitamins, pre/probiotics, medium-chain triglycerides) were excluded from the review. During the review process, articles related to food allergies or oral allergies that did not discuss complementary feeding were excluded. Additionally, articles were excluded if they focused on other microbiome-related factors, such as breastfeeding, formula feeding, or mode of delivery, without addressing complementary feeding.

## 3. Results 

### 3.1. Stepwise Process for Selecting Articles to Review

**Figure 1:**
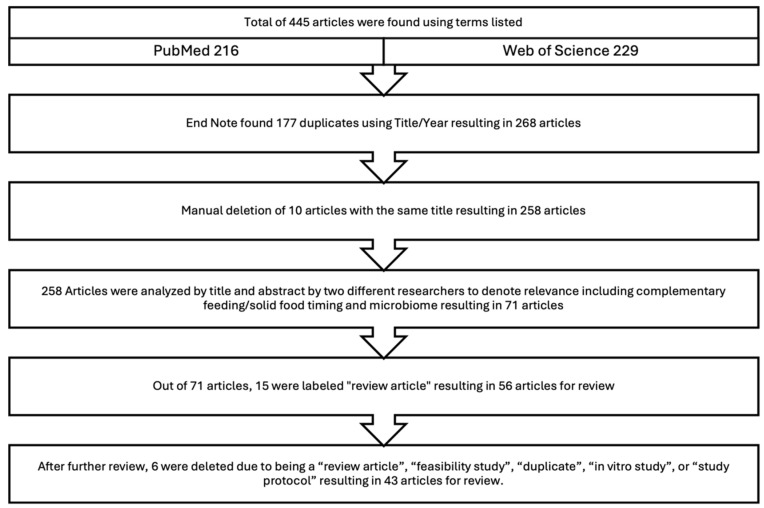
Stepwise process for selecting articles to review.

Out of a total of 445 articles, EndNote identified 177 duplicates based on title and year, leaving 268 articles. An additional 10 articles with identical titles were manually removed, resulting in 258 articles. Using titles and abstracts, the identified 258 articles were screened independently by D.N. and M.T. for relevance to the review topic, focusing on complementary feeding, complementary food timing and the microbiome, narrowing the selection to 71 articles. Among these, 15 were categorized as “review articles”, leaving 56 articles. Following an in-depth reading of the article text, 6 articles were further excluded for being review articles, feasibility studies, duplicates, in vitro studies, or study protocols, resulting in 31 articles discussing complementary foods and 13 related to complementary food timing, totaling 43 articles for this review. The stepwise approach for determining articles to review is demonstrated in Figure 1. The characteristics of the included studies and their outcomes are detailed in Table 1. Studies included in the review are found in descending order from the most to least recent publication year; for those published in the same year, studies are ordered alphabetically. Studies evaluating solid food timing are included in a second section of the table.

### 3.2. Solid Food Timing

#### 3.2.1. Microbial Diversity

Alpha-diversity was assessed using both UniFrac and the Shannon diversity index; UniFrac can be used to determine whether communities are significantly different [54], while the Shannon index weights the numbers of microbial species by their relative evenness data [55]. Alpha-diversity tended to be lower in infants preceding solid food introduction and increased throughout infancy until 2 years of age [42,49,50,52] with only one study reporting that beta-diversity of the infant gut microbial community increased during the first 6 months of life when measured according to UniFrac, though it was higher in formula-fed infants [44]. Parkin showed that breastfed infants introduced to solid foods before 5 months of age had greater alpha diversity compared to infants who had solid foods introduced after 6 months [41]. The impact of the timing of solid food introduction on diversity was conflicting; one study reported no impact on Shannon’s diversity index in infants weaned before or after 4 months [45], while another reported that infants introduced to complementary foods before 3 months had a higher Shannon diversity [46]. Vallès noted that the Shannon index increased from 3 months (before solid food introduction) to 7 months (after solid food introduction) and then decreased afterward until 1 year of age [53]. Pannaraj reported rapid maturation of the infant stool microbiota in infants with early solid food introduction before 4 months of age [52]. Two studies reported no significant differences in microbiome diversity related to solid food timing. Raspini reported no differences associated with the early introduction of solid foods, defined as introduction before 4 months [24]. The Shannon diversity index and richness (i.e., number of bacterial taxa per infant sample) were higher in infants who had solid foods introduced between 3–6 months compared to infants with a later solid food introduction; however, after adjusting for demographics, this difference was no longer significant [33].

#### 3.2.2. Microbial Taxa 

Infant age and the timing of solid food introduction were both found to play key roles in the development of the gut microbiota. In the first 6 months of life, *Bifidobacteriaceae* is the predominant family in most infants [44,50], followed by *Enterobacteriaceae* [44]. Near the time of solid food introduction, *Bifidobacterium* spp. may increase [31]; however, the impact of solid foods on *Bifidobacterium* was inconsistent, with many studies reporting a decrease [36,42,50]. With age, Pannaraj observed an increase in *Bifidobacteriaceae* [52], while Bergstrom found an increase only in *Bifidobacterium adolescentis* and *Bifidobacterium catenulatum* [36]. Other studies reported that *Bifidobacterium* maintained a relatively stable abundance during complementary feeding [13,33,39]. After the introduction of solid foods, the predominant families shifted from *Bifidobacterium* to *Lachnospiraceae* and/or *Ruminococcaceae* [50]. Tanaka also reported a shift to *Bacteroidaceae* [50]; however, this is contrasted by Savage and Shi, who both reported no association between solid food and relative abundance of *Bacteroides* spp. [13,34]. *Prevotella*, *Faecalibacterium*, and *Roseburia* increased with solid food introduction [42], and Oyedemi noted the presence of species such as *Enterococcus*, *Roseburia*, and *Coprococcus* after solids were introduced in a low- to middle-resource setting [17]. 

Two studies found a positive association between solid food introduction and *Clostridium* spp. [17,33]; another study reported an increase in *Clostridium leptum* between 9 and 36 months but a decrease in *Clostridium coccoides* between 9 and 18 months [36]. Shi also reported increases in *Blautia*, *Fusicatenibacter*, *Parasutterella*, and *Akkermansia* alongside decreases in *Escherichia-Shigella* and *Prevotella* associated with solid food introduction [13]; the same authors reported a decrease in *Lactobacillus* [13], which was supported by the findings of Bergstrom [36], but contrasted with the findings of Savage, who reported no association [33]. These studies did not discuss the types of solid foods consumed during the weaning period, which may explain some of the observed variation. 

These taxa changes may be dependent on when solid foods are introduced in addition to the type of complementary foods offered and the primary liquid diet; findings by Vacca suggested that weaning after the fourth month of age increases *Ruminococcaceae* and *Faecalibacterium* compared to an early weaning pattern (≤4 months); these differences remained significant after controlling for mode of feeding [45]. Differding reported a higher abundance of *Akkermansia muciniphila*, *Lachnoclostridium indolus*, *Bacteroides*, and *Streptococcus* in infants introduced to complementary foods before 3 months of age, and a decrease in *Bilophila wadsworthia*, *Bifidobacterium*, and *Dialister succinicivorans*, trends that persisted when adjusted for breastfeeding and formula feeding [47]. After weaning, new bacterial genera including *Alistipes*, *Dialister*, *Prevotella*, *Faecalibacterium*, *Ruminococcus*, *Roseburia*, and *Eubacterium* were seen in formula-fed infants older than 5 months [48]. Additionally, *Lactobacilli* was reported to increase initially in weaning breastfed infants before decreasing slightly after the fourth month [39]. 

The effect of age on microbial taxa has other conflicting results. One study reported that the majority of bacterial taxa changes occur between 9 and 18 months after the introduction of complementary feeding [36], while Bernal described a decrease in *Enterococcus* over 2 months during the complementary feeding period [37]. In most studies, *Clostridium* increased and was found at higher levels [47,51], though this was species-dependent as *Clostridium paraputrificum* decreased [47]. *Blautia* was reportedly increased compared to adults [51] and throughout weaning [19,28]. While Ye reported that *Bacteroides* was found at higher levels in 12-month-old infants than in adults [51], and Brink reported an increase in the proportion of *Bacteroides* at 12 months [26]. Differding found a decrease associated with early complementary food introduction [47], and Coker described decreases over the first year of life [19]. The effect of complementary feeding on *Ruminococcus* concentration was also conflicting, as Differding described a decrease associated with early complementary feeding [47] and Coker reported an increase during the first year [19]. Other increased taxa included *Parabacteroides* and *Roseburia* [47] and *Faecalibacterium* [19]. Decreased taxa included *Enterobacteriaceae* and *Dorea formicigenerans* [47] and *Bifidobacterium*, *Escherichia coli*, *Staphylococcus*, and *Klebsiella* [19]. Both Coker and Brink reported a decrease in *Bifidobacterium* by 12 months [19,26]. Finally, during post-weaning, Kujawska described a decrease in the proportion of *Bifidobacteria* across all breastfed samples [28]. 

#### 3.2.3. Gut Microbial Metabolites, Including Short-Chain Fatty Acids (SCFAs)

Studies have also discussed the production of SCFAs—fermentation products with a critical role in intestinal physiology and immunity—related to solid food timing. Bridgman reported that factors associated with a lower SCFA concentration included lower gestational age at birth, birth via cesarean section, not receiving breast milk, and solid food intake [43]. The transition to semi-solid food increased SCFAs in 7 to 9-month-old infants [20], and the addition of complementary foods showed stronger correlations to metabolites in the infant gut microbiome [16]. At 9 months compared to 18 and 36 months, more lactic acid-producing bacteria were found [36]. In a study by Differding, infants had higher SCFA concentrations of total SCFA, butyric acid, propionic acid, and acetic acid at 12 months when solid foods were introduced at or before 3 months compared to later introduction [47]. A correlation between complementary feeding and metabolites such as butyric acid was also reported by Shi [13] and Oyedemi [17]. Butyric acid increased from the start of complementary foods to 12 months in infants consuming meat, but not those consuming dairy [15]. Lastly, although seen prior to solid food introduction during the first year of life, 4-hydroxyphenyllactic and indolelactic acid almost disappeared completely by 24 months [25].

### 3.3. Dietary Intake

Before the introduction of complementary foods, the infant diet consists of breastmilk, infant formula, or a combination of the two. The liquid diet pattern during infancy has been shown to significantly impact the gut microbiome [56,57]. While the scope of this review focuses on complementary foods and the gut microbiota of infants, variations in reporting and controlling for the mode of feeding limit the generalizability of findings between liquid diets. The mode of feeding is discussed where applicable related to nutrient intake. 

#### 3.3.1. Microbial Diversity

Many studies found that dietary patterns and food choices impacted alpha diversity and taxonomic differences. McKeen and Homann reported that in general, complementary foods and dietary diversity were both associated with increased microbial richness, particularly in the early months of complementary feeding, in primarily breastfed infants [16,21]. Macronutrients, such as protein and fiber [35] and fats and oils, increased both next-day alpha- and beta-diversity, as well as generally greater microbial diversity in breastfed infants from urban slums in Mumbai [27]. Alpha-diversity was also significantly lower in infants fed via baby-led weaning compared to a control group with conventional weaning and higher with the intake of breads and cereals, fruits and vegetables, and fiber compared to other food groups. In this cohort, the mode of feeding was more variable, including breastfed, formula-fed, and mixed-fed infants [32]. Other studies found no differences in microbial diversity after the introduction of solid foods, such as legumes [29] and dairy [30]. Bruce found no associations between diet diversity scores and patterns and microbial alpha diversity [14] regardless of the mode of feeding, reflecting the need for further research to elucidate which foods and dietary patterns may be influencing microbial alpha diversity. Homann demonstrated a positive association between dietary diversity and *Bifidobacteria* and a negative association with *Veillonella* in breastfed infants [21], while Oyedemi reported a strong influence of diet and the mode of feeding on taxonomic differences in gut microbiota [17]. Homann also reported stabilization of the gut microbiome by high daily dietary diversity [21]. 

#### 3.3.2. Macronutrients

Carbohydrates are a common first food during infancy. The carbohydrate index showed no significant association with the Shannon index, though rye bread was positively associated with the Shannon index; additionally, *Lachnospiraceae* and *Ruminococceae*, which utilize complex carbohydrates, were negatively affected by breastfeeding duration [35]. Infants who received cereal with a higher ratio of complex:simple carbohydrates had higher fecal counts of *Bifidobacterium* and lower counts of *Bacteroides* in exclusively formula-fed infants [37]. The type of carbohydrate consumed may also have an impact on the infant gut microbiota; in a cohort of breastfed, mixed-fed, and formula-fed infants, *Clostridium* and *Bacteroides* were associated with a higher consumption of total sugar, and a high amount of free sugar was associated with a higher *Parabacteroides* genus [11]. Bernal, however, reported that fecal counts of *Bifidobacterium*, *Lactobacillus*, *Enterobacteriaceae*, *Clostridium*, and *Bacteroides* did not differ in infants receiving a higher digestible starch cereal versus a cereal higher in dextrins and total free sugar, though this study was conducted exclusively in formula-fed infants [37]. Plaza-Diaz noted that after the introduction of 0% whole grain or 50% whole grain cereals in formula-fed infants, *Veillonella* increased, and *Enterococcus* decreased in both groups; *Actinobacteria* and *Bifidobacterium* decreased after 0% whole grain cereal was given, and *Lachnoclostridum* and *Bacteroides* increased with the introduction of 50% whole grain cereal [23].

The findings of the association between fat and microbial diversity were conflicting, though the populations studied were variable. In breastfed infants living in Mumbai, fat and oil consumption were associated with next-day alpha and beta diversity, as well as greater microbial diversity with higher fat intake from complementary foods [27]. However, Laursen reported a negative correlation between fat consumption and Shannon’s index in Danish infants [35]. Oil and fat consumption was also associated with a higher *Lactococcus*:*Anaerococcus* log ratio than infants who did not consume additional oil and fat [27]. Fat and oil consumption in formula-fed infants was not discussed in any included study.

Protein and the gut microbiota were evaluated in multiple studies. Considering microbial taxa, Smith-Brown reported that the intake of vegetarian proteins (soy, nuts, seeds, and pulses) was positively correlated with *Clostridium* in infants receiving breastmilk [30]; however, Ordiz reported no difference in alpha diversity associated with legume supplementation [29]. Laursen reported a positive association between protein intake and the Shannon index [35]. Findings from the same study suggested negative associations of protein intake with *Bifidobacterium*, *Enterococcaceae*, and *Lactobacillaceae*, and positive associations with a cluster formed of *Erysipelotrichaceae*, *Peptostreptococcaceae*, *Lachnospiraceae*, *Clostridiaceae*, *Sutterellaceae*, and *Ruminococcaceae*; the authors suggested that protein, along with fiber, may be the major drivers of microbial changes in breastfed infants [35]. 

#### 3.3.3. Dairy

According to studies included in this review, dairy foods during complementary feeding can impact a variety of taxa present in the infant gut microbiome. Smith-Brown reported that dairy intake was negatively associated with *Bacteroides* in 6–24-month-old infants who received any breastmilk at the time of complementary food introduction, but dairy was not associated with microbiota beta diversity [30]. In a study by Bruce, a dietary pattern categorized by high dairy and formula intake compared to breastmilk was a strong predictor of variation in the gut microbiome and serum metabolome of 12-month-old infants, along with a higher abundance of *Blautia* [14]. Dairy was also associated with a lower abundance of *Lactobacillus*, *Bifidobacterium*, *Veillonella*, and *Megashpaera* spp. in the same population [14]. Tang found that consumption of dairy during complementary feeding by formula-fed infants increased *Akkermansia* in the infant gut microbiome, but did not increase butyric acid [15]. Cheese was also positively associated with alpha diversity in breastfed infants [35]. 

#### 3.3.4. Meat

Another food examined by studies in this review was meat. Unbalanced meat consumption resulted in more *Lactococcus*, *Granulicatella*, and *Acinetobacter* at 12 months in infants; this analysis did not distinguish if results differed between breastfeeding versus formula feeding [22]. Qasem reported that the introduction of meat did not decrease the proportion of *Bifidobacteriaceae* in exclusively breastfed infants [34]. In a study of 5–9-month-old breastfed infants, Krebs reported that Actinobacteria, genera *Bifidobacterium*, *Rothia*, and *Lactobacillales*, and Firmicutes did not change over time when meat was provided to the infant; additionally, the mean abundance of *Clostridium group XIVa* was increased by 40% in infants receiving meat compared to 10% in the iron-fortified cereal group; *Bacteroidales* was less abundant in the meat group comparatively [38]. Contrasting the findings from introducing dairy, Tang reports that *Akkermansia* was decreased in formula-fed infants consuming a meat-based diet [15].

Studies that assessed associations of microbial diversity with meat intake were conflicting. Leong reported no statistical significance in alpha diversity associated with meat intake in a cohort of both breastfed and formula-fed infants [32]. However, other groups reported that meat intake increased alpha diversity [35] and gut microbiota richness in both breastfed and formula-fed infants [15,34,35].

#### 3.3.5. Iron

Iron is a nutrient of concern in older infants due to their relatively high iron requirements; as breastmilk has low concentrations of iron, complementary food choices, including iron-fortified cereals and meat, are critical to meeting iron intake goals. Studies included in this review reported that gut microbial richness increased after the provision of iron-fortified cereal and meat in breastfed infants, suggesting that iron may be a driving factor of richness during complementary feeding [34]. Qasem also reported a decrease in *Bifidobacteriaceae* associated with the introduction of iron-fortified cereal in breastfed infants, but not meat [34]. In a study by Krebs, iron-fortified cereal compared to a combination iron-zinc cereal and a meat group in breastfed infants reflected a decreased abundance of *Bifidobacterium* and *Rothia*, and *Lactobacillales* and a higher abundance of *Bacteroidales*; in the same study, dietary iron was correlated with relative abundance of Enterobacteriaceae, further suggesting that iron plays a role in the shaping of the infant gut microbiota [38]. Excessive meat consumption was linked to overgrowth of iron-dependent bacteria and bacterial iron metabolism in a breastfed/formula-fed cohort [22]. Total dietary iron was significantly correlated with Enterobacteriaceae [38], which may indicate that regardless of iron source, the gut microbiota is significantly influenced by iron consumption during complementary feeding. 

#### 3.3.6. Fiber

Fiber was another common dietary component included in the studies. Similar to their findings with protein, fiber was positively associated with Shannon’s index at 9 months of age [35] and alpha diversity at 12 months of age [32] in breastfed and formula-fed infants. *Bifidobacteriaceae*, *Enterococcaceae*, and *Lactobacillaceae* were negatively associated with fiber intake at 9 months, while *Eubacteriaceae*, *Pasteurellaceae*, *Prevotellaceae*, *Veillonellaceae*, and *Fusobacteriaceae* were positively associated with fiber intake [35]; as family food, or foods typically consumed in later infancy, such as table foods prepared for the entire family, which may be higher in fiber, correlated to similar changes in the gut microbiota, Laursen suggested that fiber may be a major driver of microbial taxa changes during complementary feeding [35]. Other taxa associated with higher fiber intake in infants regardless of mode of feeding were *Faecalibacterium* and *Coproccus* [11].

#### 3.3.7. Other Dietary Components

Various other dietary components were discussed in relation to the gut microbiota across these studies. Shi reported that dietary supplementation in breastfed infants with vitamins, fish oil, and probiotics, along with fruits, during complementary feeding was linked to changes in *Sellimonas*, *Peptostreptococcus*, *Parasutterella*, *Parabacteroides*, and *Akkermansia* [13]. However, a study by Bruce returned no effect of animal or plant foods on variation in the gut microbiome during the first year of life [14]. A study supplementing bovine colostrum and egg during complementary feeding in breastfed infants reported that *Streptococcus thermophilus* was found in higher quantities in the supplemented group, but no difference in fecal bacterial profiles was detected in Malawian infants [18]. When supplemented with prebiotic galacto- and fructo-oligosaccharides, *Bifidobacteria* significantly increased in formula-fed infants [40]. Lastly, Oesterle reported that exposure levels to adverse xenobiotics and mycotoxins were high during complementary feeding in Nigerian infants compared to breastfeeding; mycotoxins correlated with *Streptococcus* in the infant gut, while xenobiotic exposure was positively correlated with *Blautia* and *Romboutsia*, and negatively correlated with *Escherichia-Shigella* in infants [12]. 

## 4. Discussion 

Multiple studies showed that alpha diversity tends to increase after solid food introduction, with a mixed impact on alpha diversity if foods were introduced “early” (prior to 4 months) [58]. Alpha- and beta-diversity are both key measures used to describe microbial variation within and between individuals/groups, respectively. As infants transition to solid foods from the liquid diet, an increase in diversity is expected and reflects the maturation of the gut microbiota [10]. Additional research is required to clarify which food types have a greater influence on the alpha diversity of the infant gut microbiota and whether the influence is positive or negative. Varying opinions were expressed on how the early introduction of solid foods might alter microbiota composition, with numerous genera linked to solid food introduction. In the first 6 months of life, *Bifidobacteriaceae* is the predominant family, but this tends to shift to *Lachnospiraceae* and *Ruminococcaceae* along with *Clostridium* spp.; the association of complementary feeding and microbial taxa requires further elucidation. Overall, several trends were observed linking dietary diversity and microbial richness, but there is a lack of research on the specific effects of types of complementary foods or food groups. Dietary information was collected for some studies; however, there were no controlled feeding trials included in this review. Inconsistencies in reporting and controlling for mode of feeding, including types of formula, add further noise when considering the impact of feeding patterns on the infant gut microbiome.

Short-chain fatty acids play a key role in human health, with findings generally supporting health benefits such as anti-inflammation, immunoregulation, and cardiometabolic protection [59,60]. In this review, butyric acid concentration increased with solid food introduction; while in adults, some studies found a higher fecal butyrate concentration was associated with worse metabolic outcomes [61], this relationship has not been well studied in infants [47]. In one study, 4-hydroxyphenyllactic and indolelactic acid had disappeared almost completely by 24 months. Bifidobacterium species promoted by breastmilk intake have previously been shown to increase both 4-hydroxyphenyllactic and indolelactic acid [62]. 4-hydroxyphenylactic acid has been shown to decrease reactive oxygen species, suggesting it may function as an antioxidant [63]. Indolelactic acid may play a role in intestinal inflammation and modulate the gut microbiome [64]. Evaluation of health outcomes associated with this decline is needed before conclusions can be drawn. 

Though outside of the scope of this review, the complementary feeding period is a critical stage in an infant’s development for influencing health outcomes, such as growth trajectories. Rapid weight gain during infancy has been associated with childhood obesity [1], with changes in the gut microbiome emerging as a contributing cause [6]. Other health outcomes with established impacts on infant gut microbiota development include allergic disease and immune function; however, the impact of food groups and dietary patterns during complementary feeding on these processes has not yet been established [65]. Despite the established implications of the gut microbiome on health outcomes in adults, a knowledge gap exists in the literature examining the influence of complementary feeding on outcomes in infants. Future research to describe the relationship of complementary foods and the gut microbiota should focus on the impact of feeding patterns and food choices on microbial diversity and maturation, including their effect on infant health outcomes.

The current evidence included in this review has several limitations. Before solid food introduction, the infant diet consists of formula or breastmilk. Although this review did not examine research preceding the introduction of complementary feeding, baseline study data often reflected the infant liquid diet; as the feeding type has a profound impact on the early establishment of the gut microbiome [56], the mode of feeding should be considered when evaluating changes in the gut microbiota during complementary feeding. Many studies in this review did not evaluate individual dietary components in relation to the gut microbiome. As each study examined different microbes and taxa, comparing the impact of dietary interventions across studies was difficult. Additionally, though the definition of “early” complementary feeding is generally recognized as introduction before 4 months of age [58], studies in this review used multiple age cutoffs, increasing the difficulty of determining the impact of early complementary feeding on microbial diversity and taxa. Study design and duration, global location, and sample size varied across the included studies, which likely influenced results and reported outcomes. Not all studies included follow-up of study participants, limiting the ability to assess the persistence of observations from complementary feeding into later childhood. Discussion of antibiotic and pre- and probiotic usage, history of the mode of feeding, including the brand of formula, and maternal microbiome are other limiting factors of the reviewed literature. Quantified dietary intake data were inconsistent across studies, and differing methodologies for microbial sequencing and bioinformatic/biostatistical analyses further limit the ability to compare results between publications due to the potential for false positive and false negative results, limiting the generalizability of reported findings. 

## 5. Conclusions

The complementary feeding period, which spans from ~6 to 24 months of age, is a critical phase when infants begin consuming solid foods, triggering shifts in the gut microbiome that may have lasting impacts on infant outcomes and long-term microbiome development. The current review underscores the need for targeted research to isolate the effects of solid foods on the gut microbiota and subsequent infant outcomes. The complementary feeding period offers a unique opportunity to shape the gut microbiome, potentially influencing outcomes like growth trajectories, obesity risk, immune function, and allergic disease development. However, recommendations and conclusions have been hindered by limitations of methodology, including study design, inconsistent definitions of early complementary feeding, lack of dietary intake data, and variability in microbiome profiling methods. Future research should utilize standardized approaches to define complementary feeding timelines and evaluate individual dietary components associated with infant gut microbiome development. Longitudinal studies with continued follow-up into later childhood are critical to understanding the persistence and implications of early dietary-microbiome interactions. Addressing these gaps will help clarify the role of complementary feeding in optimizing infant gut microbiome development and improving long-term health outcomes. Future research could focus on answering questions such as: how do specific foods introduced during complementary feeding impact the gut microbiome, what role does the liquid diet and timing of solid food introduction play in conjunction with the complementary foods, and what relationship exists between the gut microbiome during complementary feeding and infant health outcomes (e.g., growth)?

## Figures and Tables

**Table 1:** Characteristics of studies included in this review.

Reference	Study Population Size (N)	Study Type	Infant Age Range (Months)	Study Focus	Outcomes
Complementary Foods and Infant Gut Microbiota					
Mokhtari P (2024) [11]	105	Cross-Sectional Study	0–6	Associations of early life nutrition and infant gut microbiome in Latino mother-infant pairs.	- *Clostridium* and *Bacteroides* were associated with higher consumption of total sugar. - High amount of free sugar associated with higher *Parabacteroides*. - *Faecalibacterium* and *Coproccus* were associated with higher total insoluble fiber intake.
Oesterle I (2024) [12]	11	Longitudinal Pilot Study	1–12	How xenobiotic profiles and gut microbiome are changed as complementary feeding is initiated in Nigerian infants.	- With introduction of complementary foods, infants became more exposed to xenobiotics. - Molecules of plant-based origin became elevated as complementary foods were introduced. Compounds in the stool-like tricetin were positively correlated with the genus *Blautia*, whereas vulgaxanthin was negatively correlated with *Escherichia-Shigella*.
Shi Y (2024) [13]	200	Cohort Study	3–8	Impact of complementary feeding on infant growth and health outcomes.	- *Bifidobacterium*, *Escherichia–Shigella*, *Streptococcus*, and *Bacteroides* maintained similar abundance throughout weaning. - During weaning, increases were observed in *Blautia*, *Fusicatenibacter*, *Parasutterella*, and *Akkermansia*. - During weaning, decreases were observed in *Lactobacillus*, *Escherichia–Shigella*, *Coprobacillus*, and *Prevotella*.
Bruce CY (2023) [14]	182	Cross-Sectional Study	12	Association between diet, gut microbiota, and serum metabolome in South Asian and White European infants.	- High adherence to formula and dairy was the strongest predictor of variation in the gut microbiota and serum metabolome of infants. - Only the formula and dairy dietary pattern, and not animal foods or plant foods consumption, explained variation in the gut microbiotas, regardless of the types of solid foods consumed in the first year of life. - No associations between diet diversity scores, diet patterns, or gut microbial alpha diversity estimates. - Milk diet pattern did not explain variation in the gut microbiota. - *Lactobacillus*, *Bifidobacterium*, *Veillonella*, and *Megasphaera* spp. were less abundant in infants with high vs. low formula and dairy adherence.
Tang M (2023) [15]	64	Randomized Control Trial	5–12	Meat- vs. dairy-based complementary foods on gut microbiota and its relation to growth.	- Infants consuming a meat-based diet had a higher gut microbiota richness. - *Akkermansia* was increased in the dairy group and decreased in the meat group. - Butyric acid increased from 5 to 12 months in the meat group but not dairy. - Dairy group had a significant increase in WLZ. - *Bifidobacterium* and *Roseburia* had a negative association with the increase in WLZ
McKeen S (2022) [16]	25	Randomized Control Trial	4–12	Characterize changes in healthy infant gut microbiome composition, metagenomic functional capacity, and metabolites over the complementary feeding period.	- Infant gut microbiome increased in alpha diversity with the addition of complementary foods along with stronger correlations to metabolites. - Sequences assigned to taxonomy and gene functions were correlated with aqueous metabolites, while taxonomic assignments correlated to lipid metabolites between 9–12 months of age.
Oyedemi OT (2022) [17]	28	Longitudinal Cohort Study	0–12	Investigate the gut microbiota in a cohort of Nigerian infants within the first year of life.	- *Clostridium*, *Enterococcus*, *Roseburia*, and *Coprococcus* species were observed once the infants commenced weaning. - The diet strongly influenced taxonomic difference in the gut microbiota between Nigerian infants along with butyrate production, which was not affected by the birthing method.
Bierut T (2021) [18]	267	Randomized Control Trial	9–17	Determine Bovine Colostrum/egg ability to reduce linear growth delay.	- No difference in the 16S configuration of the fecal microbiota between children receiving Bovine Colostrum (BC)/egg and control. - *Streptococcus thermophilus* was found in higher quantities in children who received BC/egg.
Coker MO (2021) [19]	229	Cohort Study	1–12	Evaluate the longitudinal effect of delivery mode and infant feeding on the gut microbiome.	- Over the first year of life, there was an increase in the species of microbiome such as *Blautia*, *Ruminococcus*, and *Faecalibacterium*, while bacteria such as *Bacteroides*, *Bifidobacterium*, *Escherichia coli*, *Staphylococcus*, and *Klebsiella* decreased over time.
Conta G (2021) [20]	1	Longitudinal Study	3–9	Follow the infant fecal microbiota and metabolome change through the first year.	- Transition to semi-solid food was characterized by an increase in short-chain fatty acids, the disappearance of HMOs, and the shift within the Firmicutes phylum.
Homann CM (2021) [21]	24	Longitudinal Cohort Study	0–14	To understand the impact of solid food introduction in early life on the gut microbiome.	- Dietary diversity was positively associated with microbial richness and diversity. - The extent of change in community structure in the introductory period was negatively associated with dietary diversity. - The gut microbiome was stabilized by high daily dietary diversity. - Dietary diversity was positively associated with *Bifidobacterial* taxa and negatively associated with *Veillonella*.
Hose AJ (2021) [22]	1133	Cohort Study	4–12	Assess feeding patterns and relation to asthma risk and the gut microbiome at school age.	- Unbalanced meat consumption (UMC) influenced the composition of the gut microbiome at 12 months with more *Lactococcus*, *Granulicatella*, and *Acinetobacter*. - UMC increased growth of iron-scavenging bacteria, such as Acinetobacter.
Plaza-Diaz J (2021) [23]	43	Randomized Control Trial	4–7	Effect of different cereals differing in whole grain and sugar content on the microbiome.	- *Veillonella* increased and *Enterococcus* decreased in both 0% whole grain (0-WG) and 50% whole grain (50%-WG). - Actinobacteria and *Bifidobacterium* decreased with the 0-WG. - *Lachnoclostridium* and *Bacteroides* increased in the 50-WG. - *Proteobacteria* and *Escherichia* were lower in 50-WG.
Raspini B (2021) [24]	61	Longitudinal Prospective Observational Study	0–12	Explore the prenatal and postnatal factors influencing the infant gut microbiome.	- No statistically significant difference in the microbiome with the early introduction of solid foods (before 4 months of age).
Sillner N (2021) [25]	42	Randomized Control Trial	0–24	To investigate the effect of probiotics on the microbiome in formula compared to control formula and breast milk in healthy neonates.	- 4-hydroxyphenyllactic and indolelactic acid almost disappeared completely after the introduction of solid food (24 m).
Brink LR (2020) [26]	210	Cohort Study	3–12	Investigate impact of fecal neonatal diet on the microbiome during the first year of life.	- Proportion of *Bacteroides* increased and *Bifidobacterium* decreased at 12 months of age.
Huey SL (2020) [27]	53	Cross-Sectional Study	10–18	Describe the diversity and composition of the gut microbiota in infants living in Mumbai urban slums to determine how nutritional status, complementary foods, feeding practices, and micronutrients are associated with gut microbiota.	- Consumption of fat and oils the previous day was associated with alpha and beta diversity. - The *Lactococcus/Anaerococcus* log ratio was significantly higher among children who consumed oils and fats compared to those who did not consume oil and fats. - Higher fat intake from complementary foods reflect dietary quality, with an increase in Firmicutes and greater microbial diversity.
Kujawska M (2020) [28]	9	Cohort Study	1–18	How Bifidobacterium longum adapts to the changing nutritional environment.	- During post-weaning, proportions of *Bifidobacteria* across all breast-fed samples decreased. - The levels of bacteria detected by members of the *Clostridium* cluster XIVa started to increase during weaning from 0.25% to 18.2%. - Strong link between infant diet and *B. longum* diversity. - Strains of *B. Longum* at different dietary stages show genomic adaptations to specific substrates.
Ordiz MI (2020) [29]	236	Randomized Control Trial	6–12	Determine if infants given daily legume supplement had alteration in 16S configuration of fecal microbiota.	- Alpha diversity of the fecal microbiome was unchanged after initiation of legume supplementation. - No alteration was seen in 16S configuration occurring from cowpea or common bean vs. the control group: cooked corn and soybean flour.
Smith-Brown P (2019) [30]	50	Cohort Study	6–24	Determine associations between food, body composition, and fecal microbiota during complementary feeding period.	- Dairy intake was negatively correlated with *Bacteroides*. - Dairy intake was not associated with beta diversity of microbiota. - Intakes of vegetarian proteins (soy, nuts, seeds, and pulses) were positively correlated with clostridium.
de Muinck EJ (2018) [31]	12	Cohort Study	0–12	Analyze fecal specimens during the first year of life to provide insight into the human gut colonization process.	- Roughly near the time of introduction of solid foods there is an increase in *Bifidobacterium* spp. and a decline in several groups within the phylum Firmicutes. - A trend demonstrated a period of accelerated convergence of GI microbiota from day of life 60 to 130, followed by a period of increasing divergence until day 200, coinciding with the introduction of solid food.
Leong C (2018) [32]	74	Cohort Study	7–12	Determine if baby-led approach to complementary feeding, which encourages early introduction of adult-type diet, results in alterations of gut microbiome.	- Alpha diversity was significantly lower in infants who were fed via baby-led weaning at 12 months of age. - No significant difference between control and baby-led weaning infants in abundances of *Bifidobacteriaceae*, *Enterobacteriaceae*, *Veillonellaceae*, *Bacteroidaceae*, *Erysipelotrichaceae*, *Lachnospiraceae*, or *Ruminococcaceae* at 7 or 12 months of age. - Genus *Roseburia* was less prevalent in baby-led weaning infants at 12 months. - The intake of “breads and cereals”, “fruits and vegetables”, and “dietary fiber” at 7 months of age were all positively and significantly associated with alpha diversity at 12 months of age. No statistical significance was seen in alpha diversity in relation to “infant milk” and “meat” intake.
Savage JH (2018) [33]	323	Randomized Control Trial	3–6	Determine association between diet during pregnancy and infancy and the infant intestinal microbiome.	- The Shannon diversity index and richness were higher among infants who had solid foods introduced by the time the stool was collected (3–6 months) compared to infants who did not start solid foods by this time. However, after data was adjusted for demographics, this was no longer significant. - No association between solid food and relative abundance of *Bacteroides* spp., *Bifidobacterium* spp., *Lactobacillus* spp., or *Clostridia* spp. in unadjusted analyses. However, after adjusting for demographics, solid food introduction was positively associated with *Clostridia* spp.
Qasem W (2017) [34]	87	Randomized Control Trial	4–7	Determine the impact of iron-rich complementary foods on the infant gut inflammation and microbiota.	- Gut microbiota richness increased after the introduction of meat or iron-fortified cereal with fruit. - Across feeding groups, including iron-fortified cereal and iron-fortified cereal with fruit or meat, there was a similar abundance of bacterial phyla and families. - *Bifidobacteriaceae* declined from 51% to 37% after the introduction of iron-fortified cereal but was unchanged after meat. - Bacteroidetes increased with the introduction of complementary feeding across all feeding groups; however, this was not significant.
Laursen MF (2016) [35]	227	Cohort Study	9–18	Determine the influence of maternal obesity on the infant gut microbiome, along with analyzing complementary feeding on the development of gut microbiota.	- *Bifidobacteriaceae*, *Enterococcaceae*, and *Lactobacillaceae*, was negatively associated with fiber and protein intake. - *Erysipelotrichaceae*, *Peptostreptococcaceae*, *Lachnospiraceae*, *Clostridiaceae*, *Sutterellaceae*, and *Ruminococcaceae* formed a cluster positively associated with protein intake, but *Bifidobacteriaceae* was negatively associated with protein intake. - *Eubacteriaceae*, *Pasteurellaceae*, *Prevotellaceae*, *Veillonellaceae*, and *Fusobacteriaceae* were all positively associated with fiber intake. - Family food correlated negatively with *Bifidobacteriaceae* and *Sutterellaceae* abundance and positively with *Lachnospiraceae* and *Enterococcaceae* abundance, suggesting that progression from early infant food to family foods with higher protein and fiber is the major driver of gut microbial changes during late infancy. - At 9 months of age, protein and fiber intake was significantly associated with a positive Shannon index, while fat was negatively correlated with the Shannon index. - No significant association was seen between the carbohydrate index and the Shannon index. - Both microbial richness and evenness were affected by the complementary diet. Foods such as cheese, meat, and rye bread were positively associated with alpha diversity. The remaining 19 food groups did not correlate significantly with alpha diversity, indicating that foods rich in fiber and protein are the main drivers of gut microbial alpha diversity development.
Bergstrom A (2014) [36]	330	Cohort Study	9–36	Determine the relationship between nutritional parameters and measures of growth and body composition in relation to the observed gut microbiota development.	- 9-month samples clustered less closely together than the later samples and were characterized by more lactic acid bacteria and enterobacteria than samples at 18 and 36 months of age. The majority of bacterial taxa occurred between 9 and 18 months of age after the introduction of complementary feeding. - *Bifidobacterium* spp. decreased in abundance, specifically *Bifidobacterium longum* and *Bifidobacterium breve* decreased while *Bifidobacterium adolescentis* and *Bifidobacterium catenulatum* increased during 9 to 36 months of age. - *Lactobacillus* spp. and *Enterobacteriaceae* decreased between 9 and 18 months, while an increase was seen in butyrate, producing taxa *Clostridium leptum* group, *E. Halli*, and *Roseburia* spp. from 9 to 36 months. The butyrate-producing *Clostridium coccoides* group reduced between 9 and 18 months.
Bernal MJ (2013) [37]	19	Randomized Control Trial	6–12	To ascertain the colonic effects of two infant cereals with different carbohydrate profiles.	- Faecal counts of *Bifidobacterium*, *Lactobacillus*, *Enterobacteriaceae*, *Enterococcus*, *Clostridium*, and *Bacteroides* did not show any statistical differences between the two groups: cereal A (higher digestible starch and resistant starch) and cereal B (higher in dextrins and total free sugars). - Although not significant, the cereal A group had a higher *Bifidobacterium* count in all visits than infants fed with infant cereal B. Likewise, not statistically significant, but *Bacteroides* was higher in group B than A for five visits. - *Enterococcus* significantly decreased from baseline until the last visit in both groups.
Krebs NF (2013) [38]	14	Randomized Control Trial	5–9	Compare iron status in breastfed infants randomized to groups receiving complementary feeding regiments that provided iron through fortified infant cereals or meats and to examine the gut microbiota in these groups.	- Firmicutes and Bacteroidetes were most abundant in infant stool samples, however the proportion of these phyla differed within individuals over time, e.g., Bacteroidetes species were observed in some infants (one in each of iron and zinc fortified cereal and three in the meat-feeding group). In these individuals, Actinobacteria, *Proteobacteria*, or Firmicutes compensated for low Bacteroidetes. - Actinobacteria, genera *Bifidobacterium***,** and *Rothia*, along with Firmicutes, genera *Lactobacillales*, significantly decreased in abundance over time in the iron-fortified cereal group but remained unchanged in the iron- and zinc-fortified cereal and meat-feeding group. - *Bacteroidales* were significantly more abundant in iron-fortified cereal compared to iron- and zinc-fortified cereal and the meat-feeding group. - In phylum Firmicutes, median abundance of *Clostridium group XIVa* clade was increased by 40% in the meat-feeding group, but by only 10% in the iron-fortified cereal group. - Neither phylum Proteobacteria nor subgroups including genera *Escherichia*, *Klebsiella*, *Haemophilus*, and *Shigella* were associated with any food group. - Enterobacteriaceae (including *Escherichia coli*) was significantly correlated with total dietary iron.
Amarri S (2006) [39]	22	Descriptive Study	4–9	To investigate changes in gut microbiota and markers of gut permeability along with the immune system during complementary feeding in breastfed infants.	- High stool *Bifidobacteria* counts were stable throughout 5 months of complementary feeding, while counts of enterobacteria and enterococci significantly increased with age. - *Lactobacilli* increased significantly during weaning from the start of the study to the third month and then decreased slightly in the fourth month. - During the study, *Clostridium perfringens* remained below the detection limit.
Scholtens PA (2006) [40]	35	Randomized Control Trial	4–8	Test the effect of solid foods with added prebiotic galacto- and fructo-oligosaccharides (GOS/FOS) on the gut microbiota of formula-fed infants during the weaning period.	- *Bifidobacteria* significantly increased from 43% to 57% in the GOS/FOS group from week 0 to week 6, but did not change in the control group.
Timing of Complementary Foods on Infant Gut Microbiota					
Parkin K (2024) [41]	170	Longitudinal Cohort Study	6	Explore whether the effects of the timing of solid food introduction into the infant diet have differential effects on the gut microbiota in breastfed versus formula-fed infants.	- Introduction of solid foods prior to 5 months of age in breastfed infants was associated with a higher alpha diversity than introduction after 6 months, predominantly due to loss of *Bifidobacterium infantis*. - In formula-fed infants, there was a higher colonization of *Escherichia ecoli* if solid foods were introduced before 5 months of age, but no change in overall diversity was seen. - Overall age of starting solid food influenced the gut microbiome composition in breastfed infants, but not in formula-fed infants.
Bhattacharyya C (2023) [42]	25	Longitudinal Observational Study	0–12	Identify factors influencing the neonatal gut microbiome and the impact of solid foods.	- Shannon diversity index of the gut microbiome was increased after solid food introduction.
Bridgman SL (2022) [43]	647	Cohort Study	3–36	Determine the temporal association between infant fecal gut metabolites, secretory IgA, and body mass index z-score of preschool children.	- Infants born at lower gestational age, via caesarian delivery, and not breastfed had lower concentrations of total SCFA. - Solid food introduction was associated with fecal metabolites (formate, butyrate, and propionate).
Ma J (2022) [44]	62	Prospective Study	0–24	Compare the gut microbiota of healthy infants based on specific interactions of delivery modes and feeding types.	- At 30 days of life, 3 months, and 6 months, *Bifidobacterium* was the most predominant genus, followed by *Enterobacteriaceae*. - Beta diversity of the infant gut microbiome increased during the first 6 months regardless of the delivery method or the mode of feeding.
Vacca M (2022) [45]	45	Longitudinal Prospective Study	0–12	Investigate the role of nutrition in shaping microbial ratios and determine the presence/absence of specific bacterial taxa in the infant gut microbiome.	- Infants exclusively breastfed for 6 months were predominantly colonized by *Lactobacillaceae* and *Enterobacteriaceae*. - High levels of *Ruminococcaceae* and *Faecalibacterium* were primarily linked to infants weaned after four months of age. Shannon’s index did not result in a statistical significance between those weaned < 4 months of age and those who were weaned after. Infants showed a significant increase in *Ruminococcaceae* and *Faecalibacterium* if they weaned after the fourth month of age compared to if they weaned prior to 4 months.
Differding MK (2020) [46]	392	Prospective Cohort Study	0–36	Examine associations of early versus later introduction of complementary foods with the composition and diversity of gut microbiota in childhood.	- The early introduction of complementary feeds at 4 months or younger was not associated with Shannon diversity. There was no difference seen in amplicon sequence variant richness in those who had early complementary feeding. - Higher abundance of *Lachnospiraceae Blautia*, *Porphyromonadaceae Barnesiella intestinihominis*, *Streptococcaceae Lactococcus lactis*, *Veillonellaceae Veillonella*, and *Lachnospiraceae Anaerostipes* was seen with the early introduction of complementary feeds compared to *Lachnospiraceae* and *Porphyromonadaceae Barnesiella intestinihominis,* which was associated with a later introduction to complementary feeds.
Differding MK (2020) [47]	67	Longitudinal Cohort Study	3–12	Examine associations of early introduction to complementary foods with the infant gut microbiota composition and diversity, and fecal SCFA concentrations.	- Infants had higher SCFA concentrations of butyric acid and total SCFA at 12 months when complementary foods were introduced early (at or before 3 months of age) vs. later. Fecal butyric acid, acetic acid, and propionic acid were not associated with early complementary feeding at 3 months of age. At 12 months, early complementary feeding was significantly associated with higher fecal butyric acid, propionic acid, and acetic acid. - Infants introduced to complementary foods at or before 3 months of age had a higher Shannon Diversity at 3 and 12 months of age. - Complementary foods introduced at or before 3 months of age was associated with significantly higher abundance of *Akkermansia muciniphilia*, *Lachnoclostridium indolis*, Bacteroides, *Erwinia*, *Streptococcus*, and *Veillonella*, and lower abundance of *Veillonella*, *Bilophila wadsworthia*, *Erwinia*, *Bacteroides, Bifidobacterium*, *Streptococcus*, and *Dialister succinicivorans*. - By 12 months of age, early complementary food was associated with higher amounts of *Parabacteroides*, *Clostridium disporicum*, *Roseburia*, and *Veillonella*, and lower amounts of *Clostridium paraputrificum*, *Eubacterium*, *Enterobacteriaceae*, *Enterococcus*, *Eubacterium hallii*, *Bacteroides uniformis*, *Ruminococcus*, *Veillonella*, *Dorea formicigenerans*, *Bacteroides*, *Parabacteroides*, *Alistipes*, *Lachnoclostridium*, and *Lachnoclostridium indolis*.
Ku HJ (2020) [48]	27	Longitudinal Study	0–17	To understand compositional changes in the gut microbiome from infancy to childhood based on diet.	- When weaning from breastmilk to solid food, Actinobacteria decreased but Firmicutes increased. Bacteroidetes became a major phylum in the solid food group. - The new genera of bacteria observed after the introduction of solid foods included *Alistipes*, *Dialister*, *Prevotella*, *Faecalibacterium*, *Ruminococcus*, *Roseburia*, and *Eubacterium*.
Mancabelli L (2020) [49]	1035	Multi-Population Cohort Meta Analysis	0–36	Evaluate the evolution of the infant microbiota composition during the early stages of life and variations due to diet.	- Alpha diversity based on the OUT index demonstrated a significant difference between breastfed compared to formula in the 6–12-month age group, indicating that the microbiota is more likely influenced by the transition to a solid food diet that starts around 6 months of age.
Tanaka M (2020) [50]	10	Longitudinal Study	0–36	Assess the occurrence of bile acids in association with the development of the infant gut microbiome.	- *Bifidobacteriaceae* was observed in all infants in the first 6 months of life. Enterobacteriaceae was the predominant family instead of *Bifidobacteriaceae* in some infants in an earlier period. - After 6 months, *Lactnospiraceae* and *Bacteroidaceae* were more dominant than *Bifidobacteriaceae*. - Stool samples collected before solid food intake started were high in *Bifidobacteriaceae* and/or *Enterobacteriaceae*, whereas after the start of solid food was higher in *Lachnospiraceae*, *Ruminococcaceae*, and/or *Bacteroidaceae*. - Alpha diversity was low during the first 6 months and increased until 2 years of age.
Ye L (2019) [51]	98	Cohort Study	0–12	To study the abundance and change in abundance of carbohydrate-active enzymes in the gut during complementary feeding.	- *Bacteroides*, *Blautia*, and *Clostridium* were found at higher levels in 12-month-old infants compared to adults.
Pannaraj PS (2017) [52]	107	Longitudinal Study	0–12	Determine the association between the maternal breast milk and areolar skin and infant gut bacterial communities.	- Bacterial diversity in the microbiome increased with age, converging near 12 months. - As the infant aged, Actinobacteria, specifically *Bifidobacteriaceae*, increased and Proteobacteria, specifically *Enterobacteriaceae*, decreased with age. - Solid food introduction was associated with a change in the composition of the gut microbiome but not phylogenetic diversity. - The rapid maturation of infant stool microbiota was seen in those with early solid food introduction < 4 months of age.
Valles Y (2014) [53]	13	Birth Cohort	0–12	Explore the patterns of taxonomic and functional changes in the infant gut microbiome over time.	- Between 1 week and 1 year of age, there was a significant increase in taxon richness. - The Shannon index increases from 3 months of age (before introduction of solid foods) to 7 months of age (after introduction of solid foods) and decreases from 7 months of age to 1 year of age.

## Data Availability

No new data were created in this study. Data sharing is not applicable to this article.
